# Tools for the Diagnosis of Herpes Simplex Virus 1/2: Systematic Review of Studies Published Between 2012 and 2018

**DOI:** 10.2196/14216

**Published:** 2019-05-23

**Authors:** Zeeshaan Arshad, Abrar Alturkistani, David Brindley, Ching Lam, Kimberley Foley, Edward Meinert

**Affiliations:** 1 Department of Medicine University of Cambridge Cambridge United Kingdom; 2 Global Digital Health Unit Imperial College London London United Kingdom; 3 Healthcare Translation Research Group Department of Paediatrics University of Oxford Oxford United Kingdom

**Keywords:** diagnostic techniques and procedures, herpes simplex, diagnosis

## Abstract

**Background:**

Herpes simplex virus (HSV)-1 and HSV-2 are common infections affecting the global population, with HSV-1 estimated to affect 67% of the global population. HSV can have rare but severe manifestations, such as encephalitis and neonatal herpes, necessitating the use of reliable and accurate diagnostic tools for the detection of the viruses. Currently used HSV diagnostic tools require highly specialized skills and availability of a laboratory setting but may lack sensitivity. The numerous recently developed HSV diagnostic tools need to be identified and compared in a systematic way to make the best decision about which diagnostic tool to use. The diagnosis of HSV is essential for prompt treatment with antivirals. To select the best test for a patient, knowledge of the performance and limitations of each test is critical.

**Objective:**

This systematic review has summarized recent studies evaluating HSV-1 and HSV-2 diagnostic tools.

**Methods:**

Following the Preferred Reporting Items for Systematic Reviews and Meta-Analyses guidelines, selection criteria, data extraction, and data analysis were determined before the commencement of the study. Studies assessing the specificity/sensitivity of HSV-1 or HSV-2 diagnostic tools published between 2012 and 2018 were included. Quality assessment of included studies was performed using the quality assessment of diagnostic accuracy studies (QUADAS-2) tool.

**Results:**

Searches of the PubMed database yielded 264 studies; 11 studies included 11 molecular assays, and 8 studies included 19 different serological assays for the detection of HSV-1, HSV-2, or both. A greater proportion of molecular assay–based tools are being developed by commercial entities. Studies that tested molecular assays mostly focused on cutaneous and mucosal HSV infections (n=13); 2 studies focused on ocular disease, whereas only 1 study focused on the central nervous system manifestations. The Simplexa HSV 1 & 2 Direct is currently the only Food and Drug Administration–approved device for use on cerebrospinal fluid. No tools focused on prenatal screening. We also present performance metrics of tests for benchmarking of future technology. Most of the included studies had a high risk of bias rating in half of the QUADAS-2 tool risk of bias domains.

**Conclusions:**

The use of serologic tests to diagnose genital lesions is inappropriate because positive results may be due to chronic infection, whereas negative results may overlook recent infection. The incidence of acute infections is rising. As these infections present the greatest risk to fetuses, work needs to be done to prevent vertical transfer. Prenatal screening for primary infection and subsequent medical intervention will assist in lowering the rate of neonatal herpes. In conclusion, HSV diagnosis is moving away from culture-based methods to serology-based or polymerase chain reaction–based methods. Sensitive, rapid, and efficient HSV diagnostic tools should be adopted for the prevention of acute infections and neonatal herpes.

## Introduction

Herpes simplex virus (HSV)-1 and HSV-2 are DNA viruses that belong to Alphaherpesvirinae, a subfamily of the Herpesviridae family [1]. They are the causative agents in a wide range of human diseases that include oral and genital mucocutaneous lesions and some rare but life-threatening conditions such as fulminate encephalitis [2].

HSV is a very common condition, with HSV-1 and HSV-2 infection estimated to affect 67% and 11% of the global population, respectively [2]. The prevalence of HSV can be even higher in low- and middle-income countries or among certain patient subpopulations. For instance, HSV-2 prevalence in sub-Saharan countries is estimated to be as high as 53.7% among individuals aged 15 to 25 years [3]. The prevalence of HSV-2 can be used as a biomarker for the HIV epidemic because of its high association among patients with an HSV-2 infection [4].

One of the most severe manifestations of HSV is encephalitis, which can have a mortality rate up to 97% [5]. Encephalitis is a rare manifestation, which could result in about 4 to 5 cases/million population/year in developed countries [6,7]. HSV infections are very common among women of reproductive age, which can increase the risk of virus transmission from the mother to the child during birth, resulting in neonatal herpes [4]. Neonatal herpes is another severe manifestation of the disease and can cause long-term health complications requiring appropriate and reliable identification of the disease.

In addition to the prevalence and potential complications, lesions caused by HSV-1 and/or HSV-2 are nonspecific and have variable presentations. Chronic carriage necessitates the use of different laboratory testing methods appropriate to each case [8]. Currently, viral culture is the most commonly used method of diagnosis, and it is considered the gold standard method [8]. This method is limited, however, by the need of a laboratory setting, aseptic technique, and variable accuracy dependent on disease stage [8]. It also takes up to 7 days to get the viral culture results, during which time the infection may have developed further. The viral culture will have a lowered sensitivity after the first 48 hours of appearance of the symptoms and is best if administered as soon as the symptoms appear [9]. Although antigens specific to HSV-1 and HSV-2 can also be readily detected by direct immunofluorescence assay, these methods have been found to lack sensitivity in nonsymptomatic patients [8].

Other methods for HSV diagnosis include serological tests, which use blood sample to check for the disease *antibodies* [9]. These tests can take between 1 day and 3 weeks [9]. Serological tests are suitable to detect the subtype of the HSV virus (HSV-1 or HSV-2) and can detect asymptomatic patients [10]. Serology-based tests can also be used to confirm a clinical diagnosis of HSV because it is a more reliable method than clinical diagnosis [10]. It has been argued that serology-based tests are more *direct* and *economical* compared with viral cultures [10].

More modern methods used for detection of infection include techniques that detect viral DNA such as polymerase chain reaction (PCR). These methods use molecular-based assays and are widely used for the detection of infectious diseases [11]. Such methods have been found to be more sensitive and rapid and require less stringent conditions in terms of collection data [11,12]. Type-specific diagnosis is also possible using antigen detection techniques such as enzyme immunoassays targeting the glycoprotein G antigen [8].

A thorough knowledge of the performance and limitations of available tests is critical to select the most appropriate test for a patient as well as inform clinical decision making and the development of future technologies. Previous systematic reviews regarding HSV diagnostic tools reviewed only commercially available tests used to diagnose only one type of the virus (HSV-2) and only reviewed tests used in sub-Saharan Africa [13]. Another review looked at HSV diagnostic tools but limited its population only to pregnant women and neonates [14]. This systematic review summarizes recent studies evaluating HSV-1 and HSV-2 diagnostic tools. We will evaluate the study characteristics, the performance of the various tests included in the studies, and finally discuss their limitations and strengths. We will then suggest areas with unmet medical needs.

## Methods

Selection criteria, data extraction, and methods of analysis were decided before the study, in line with the Preferred Reporting Items for Systematic Reviews and Meta-Analyses (PRISMA) guidelines [15].

### Eligibility Criteria

We selected only original articles published between 2012 and 2018. We used the Population, Intervention, Comparator, Outcome (PICO) framework to highlight the review’s inclusion and exclusion criteria (Textbox 1). The population included both animal and human populations of all sex and age groups. Patients with multimorbidities were also included, for example, patients with hepatitis and/or HIV because of the possibility that HSV coexists with diseases such as HIV and hepatitis in some patient populations [16,17]. The intervention under study included diagnostic tools to detect all types of HSV-1 or HSV-2 infections including oral, genital, ocular, or central nervous system (CNS)–related infections. The elements in the PICO framework were determined as a result of a preliminary search in PubMed before the formulation of the review’s eligibility criteria. To help compare studies and their outcomes, we only included studies that reported sensitivity and/or specificity as performance indicators of the diagnostic tools [18]. We excluded book chapters, reviews and reports, and articles not in the English language. To capture the most up to date evidence, we restricted the search to the years 2012 to 2018 (Multimedia Appendix 1).

### Search Strategy and Sources of Information

The complete search strategy used to identify studies is detailed in Multimedia Appendix 1. PubMed (MEDLINE) was searched from January 1, 2012 to December 31, 2018. We only searched the PubMed database because most of the studies concerning diagnostic tools are indexed in MEDLINE [19]. We also searched the bibliographies of relevant articles and reviews that we identified in our initial scoping searches.

### Study Selection

Studies were selected first by removing duplicates from the initially identified studies, then manually screening the titles and abstracts of the studies against the eligibility criteria, and finally performing full-text reading of eligible studies. ZA and AA completed study screenings. Study records were managed using Mendeley Desktop (v1.16.3) software.

### Data Extraction

Data extraction sheets were developed before data extraction. Data extraction was completed separately by the study authors ZA and AA to increase accuracy. Items collected in the data extraction sheets were identified and finalized through iterative discussions with the study authors.

The data extraction sheets collected information on the following:

1. General study information including country of the study, HSV subtype, sample size, study design (retrospective or prospective), the name of the diagnostic test under study, as well as the comparison tools.
2. Methodological details including sample collection method, transportation/storage method, study aims, and conclusions.
3. After identification of the diagnostic test under study, the tests were categorized as follows: serology-based HSV detection assays, molecular assays for detection of HSV, or culture-based methods. Tables for each method were then created that summarized the performance of each test. Although sensitivity and specificity were our primary performance metrics, agreement (total, positive, and negative), kappa, area under receiver operator curve, negative predictive value, and positive predictive value were also recorded. Cost and time to run the test were also extracted. In addition, the Food and Drug Administration and European Medicines Agency status of each included diagnostic tool were ascertained through search of the respective organizations websites [20,21]. For studies that reported analytical performance and clinical performance, only the clinical performance results were reported in this review.

### Risk of Bias in Studies

The risk of bias of included studies was assessed using the QUADAS-2 tool [22].

**Review inclusion and exclusion criteria reported using the Population, Intervention, Comparator, Outcome framework.**

Population

- All animal and human populations, including individuals of all ages and both sexes such as neonates, infants, children, adolescents, and pregnant women.
- Individuals with multimorbidity will also be included such as patient coinfected with herpes simplex virus (HSV) and HIV, or HSV and hepatitis C.

Intervention

- All diagnostic tests for HSV-1 or HSV-2 used for any type of the infection such as oral, anogenital, ocular, or central nervous system infections.
- Diagnostic tests that detect other viruses in addition to HSV-1 and HSV-2 will not be included.

Comparator

- Not specified.

Outcome

- Sensitivity and/or specificity as performance measured of the diagnostic test.

Dates

- Database searches will be limited to the dates April 17, 2012 to December 31, 2018.

## Results

### Search Results and Study Inclusion

Following PRISMA guidelines [23], we identified 264 records from the PubMed search and an additional 30 through the bibliography search. There were no duplicates in the records identified; hence, all 294 record titles and abstracts were screened for eligibility; 235 articles were excluded based on the eligibility criteria. Full-text reading of the 59 screened articles resulted in a total of 19 full-text studies for inclusion in this systematic review (Multimedia Appendix 2). We screened the abstracts of 264 articles for relevance and found 59 records that could not be excluded based purely on reviewing the abstracts and titles and these were assessed as full texts. We subsequently excluded 40 articles if, after deeper examination, we found that there was no performance metric measured (n=8), the article did not include a diagnostic tool (n=9), study described viruses other than HSV-1 or HSV-2 (n=11), sensitivity and/or specificity were not measured (n=8), or the article was a review (n=4).

### Study Characteristics

Summaries of the 19 included studies can be found in Tables 1 and 2. The majority of studies were prospective in nature (n=12; fewer studies (n=7) relied on archived samples (Table 1). The number of studies over the last 6 years has grown, reaching its highest in 2017: from 5 studies in 2012 to 11 in 2017 (Table 1). This growth declined in 2018, with only 1 study found to be focused on HSV-1 or 2 diagnostic tools. Although the search was completed on December 31, 2018, there may be other 2018 studies that were yet to be published on the day of the search. Sample sizes within studies ranged from 60 in Al‐Shobaili et al [24] and Loughman et al [25] to 3408 in Patel et al. [26] with a median sample size being of 179 patients (Table 1). Studies investigated diagnostic tools for HSV-1/2 (n=10), HSV-2 only (n=7), or HSV-1 only (n=2), and in 1 study, the viral subtype could not be ascertained [27], so it was labelled as HSV-1/2. In addition, 58% of tests were molecular assays, whereas the remainder used serology-based HSV detection methods. No study used culture-based method as their primary method; studies did use them as a comparison method (Table 1). In terms of disease state, 2 studies investigated patients with ocular disease [27,28], 1 for CNS infection [29], and the remainder for patients with suspected mucosal and/or cutaneous lesions (Table 1). Given the heterogeneous nature of the studies included within this review in terms of sample type, collection, and patient demographics, it was not possible to draw statistical inferences or perform a meta-analysis.

The methodological characteristics of included studies are presented in Table 2. Basic demographic information was reported in 13 studies. In studies that reported participant gender (n=11), between 30% and 92% of study participants were male. Participants’ age ranged from 5 months to 85 years (Table 2).

### Diagnostic Test Performance

#### Molecular Assays

Molecular assays for detection of HSV are summarized in Table 3. A total of 11 diagnostic tools were investigated within the 11 studies (including 1 study that used both molecular and serological methods in conjunction [27], out of which 6 have received FDA approval; Table 3). Some FDA-approved tools had an approval for only some components of the test or certain uses of the test only. For example, the Viper HSV-Qx assay [34] and the BD ProbeTec HSV-Qx (HSVQx) system [40] were only approved for anogenital lesions, Luminex ARIES HSV-1 & 2 Assay [35] was approved for cutaneous of mucocutaneous lesions only; 8 study samples were collected using mucosal or cutaneous swabs (Table 3); in the remaining 3 studies, 1 collected samples through corneal scrapings or conjunctival swabs, 1 collected CSF, and 1 tears (for ocular manifestations; Multimedia Appendix 3).

**Figure 1:**
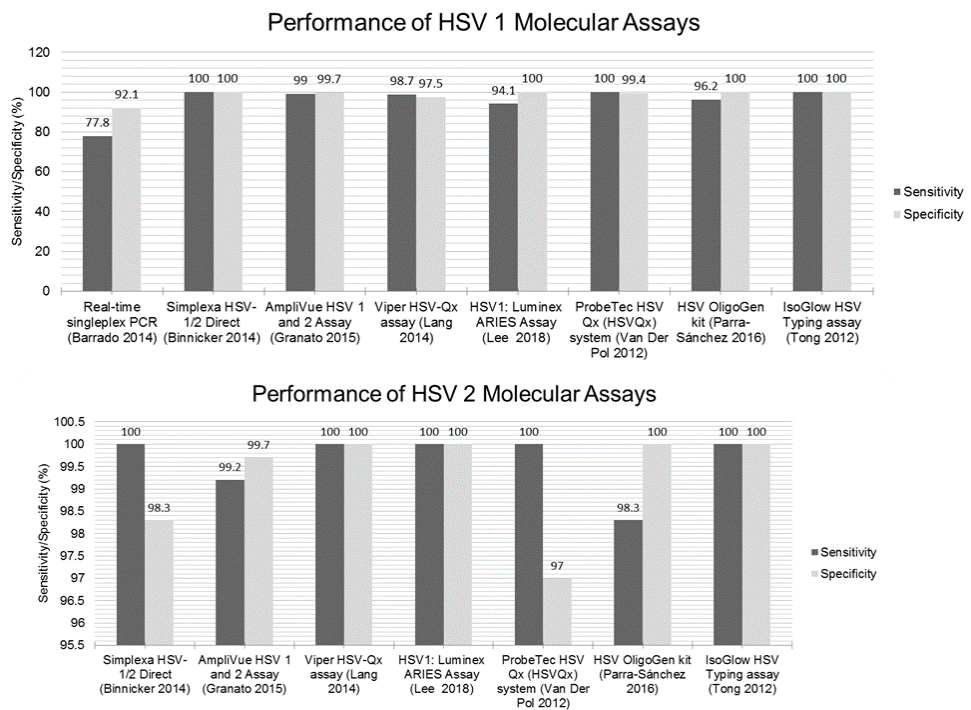
Sensitivity and specificity of molecular-based diagnostics. Because of the heterogeneous nature of studies included in this systematic review, it is not possible to draw strict conclusions with regard to performance metrics. This figure, therefore, aims solely to act as a means of visually displaying the sensitivity and specificity results of each study. HSV: herpes simplex viruses.

**Figure 2:**
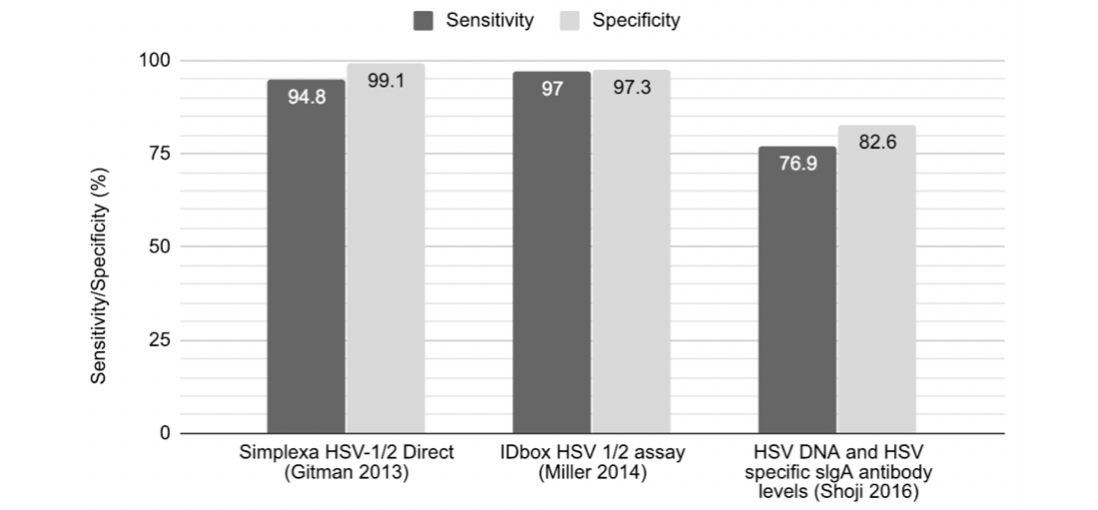
Sensitivity and specificity of molecular-based diagnostics. Because of the heterogeneous nature of studies included in this systematic review, it is not possible to draw strict conclusions with regard to performance metrics. This figure, therefore, aims solely to act as a means of visually displaying the sensitivity and specificity results of each study. HSV: herpes simplex viruses.

The performance metrics of the various molecular tests are summarized for comparison with future technologies summarized in Figures 1 and 2. One study [35] reported positive percent agreement and negative percent agreement instead of sensitivity and specificity, which is equivalent to the sensitivity and specificity measures in the absence of a standard [43]. Sensitivities for molecular methods ranged from 76.9% (27) to 100% (for a number of tools) (Figures 1 and 2). The study by Shoji et al [27] was the single study that used both a serological and molecular technique in conjunction using tears as a sample material. The study reported the lowest specificity of 82.6% using their ELIZA and PCR techniques. The second worst sensitivity was indicated by a real-time singleplex PCR tested in an academic center by Barrado et al [28]. Here, a sensitivity of 77.8% using corneal scrapings was found [28]. All other values were above 90% (Multimedia Appendix 3; Figures 1 and 2). Gitman et al [31] aimed to compare cell culture, DFA, and a laboratory-developed real-time TaqMan PCR (LDT HSV PCR) for the detection of HSV in dermal, genital, ocular, mouth, or other swab samples [31]. Conventional culture was found to have a sensitivity and specificity of 87.9 (0.768-0.943) and 99.1 (0.945-1.000), respectively. The difference when using direct immunofluorescence, TaqMan PCR, or Simplexa Direct PCR did not improve these metrics [31].

**Table 1:** Summary of included studies. Table summarizes study location, herpes subtype, sample size, study design (retrospective or prospective), and the name of the diagnostic test under study as well as the tools it will be compared with.

Study ID	Country	Study design	HSV^a^-1/2	Sample size	Disease	Test	Test type	Comparison
Al‐Shobaili et al [24]	Saudi Arabia	Prospective	HSV-2	60: 35 male and 25 female	Genital ulcer disease (genital)	Herpe Select Express Rapid Test (Focus Technologies Inc, Cypress, CA)	Immunochromatographic (serology-based HSV detection assay)	HerpeSelect 2 IgGELISA (Focus Technologies Inc, Cypress, CA); Kalon HSV-2 IgG^b^ ELISA^c^ assay (Kalon Biological Ltd, Guilford, United Kingdom); MAb-EIA^d^
Barrado et al [28]	Spain	Prospective	HSV-1	188	Suspicion of herpetic epithelial keratitis and nonherpetic corneal ulcer (ocular)	Real-time singleplex PCR^e^	Molecular assays for detection of HSV	Cell Culture- csPCR, csCC, cswPCR, csWCC
Burton et al [30]	United States	Retrospective	HSV-2	84	Veterans with hepatitis C and HSV-2 coinfection	Focus HerpeSelect Hsv-2 IgG (Focus Technologies Inc, Cypress, CA)	Serology-based HSV detection assay	Biokit HSV-2 rapid assay (Biokit United States, Lexington, MA)
Gitman et al [31]	United States	Prospective	HSV-1, HSV-2	171	Dermal, genital, ocular, mouth	Simplexa HSV 1 & 2 PCR (Focus Diagnostics, Cypress, CA)	Molecular assays for the detection of HSV	Cell culture (Diagnostic Hybrids, Athens, OH); Cytospin-enhanced DFA^f^; Real-time TaqMan PCR (LDT^g^ HSV PCR)
Granato et al [32]	United States	Prospective	HSV-1, HSV-2	1351	Cutaneous and mucocutaneous herpes infection (cutaneous)	AmpliVue HSV-1 & 2 assay (Quidel, San Diego, CA)	Molecular assays for detection of HSV	ELVIS HSV ID and D3 Typing System (Quidel DHI, Athens, OH)
Hobbs et al [33]	Kenya	Retrospective	HSV-2	198	Not specified	HerpeSelect 2 (Focus Diagnostics, Cypress, CA); Kelon ELISA (Kalon Biological, Guildford, United Kingdom)	Serology-based HSV detection assay	Same tests but with serum sample
Lang et al [34]	Canada	Prospective	HSV-1, HSV-2	276	Anogenital, oral	Viper HSV-Qx assay (BD Molecular Diagnostics)	Molecular assays for detection of HSV	LightCycler 2.0 platform (HSV-LC) (Roche Diagnostics, Basel, Switzerland)
Lee et al [35]	Singapore	Retrospective	HSV-1, HSV-2	117	Not specified	Luminex ARIES HSV-1 & 2 assay (Luminex Corp, Austen, TX)	Molecular assays for detection of HSV	Förster resonance energy transfer-based PCR assay and FTD Neuro 9 assay (Fast Track Diagnostics, Junglinster, Luxembourg)
Liermann et al [36]	Germany	Retrospective	HSV-1, HSV-2	263	Not specified	Orgentec ELISA Anti-HSV-2 IgM (Mainz, Germany); Orgentec ELISA Anti-HSV-1 IgM (Mainz, Germany); Orgentec ELISA anti-HSV-1/2 IgM (Mainz, Germany); Serion ELISA classic HSV-2 IgM IgM (Würzburg, Germany); Serion ELISA classic HSV-1 IgM (Würzburg, Germany); Serion ELISA classic HSV-1 + 2 IgM IgM (Würzburg, Germany); Orgentec ELISA Anti-HSV-2 IgG (Mainz, Germany); Orgentec ELISA anti-HSV-1 IgG (Mainz, Germany); Serion ELISA classic HSV-2 IgG IgM (Würzburg, Germany); Serion ELISA classic HSV-1 IgG IgM (Würzburg, Germany); Serion ELISA classic HSV-1 + 2 IgG IgM (Würzburg, Germany)	Serology-based HSV detection assay	HerpeSelect 1 ELISA IgG (Focus Diagnostics, Cypress, CA); HerpeSelect 2 ELISA IgG (Focus Diagnostics, Cypress, CA); Immunoblot assay recomLine HSV-1 & 2 IgG (Mikrogen, Neuried, Germany)
Liu et al [37]	China	Prospective	HSV-2	318	Not specified	gG_321580His_ ELISA test	Serology-based HSV detection assay	HerpeSelect 2 ELISA IgG (Focus Diagnostics, Cypress, CA)
Loughman et al [25]	France	Retrospective	HSV-2	60: 42 female and 18 male	Not specified	HSV-2 biochip test using reagents from the HerpeSelect® 2 IgG kit (Focus Diagnostics, CA; product EL0920G)	Serology-based HSV-2 immunoassay with biochip	LIAISON HSV-2 type specific IgG chemiluminescent immunoassay test (DiaSorin, MN)
Binnicker et al [29]	United States	Prospective	HSV-1, HSV-2	100	Central nervous system infection due to herpes simplex virus (CNS)	Simplexa HSV 1 & 2 Direct (Focus Diagnostics, Cypress, CA)	Molecular assays for detection of HSV	Roche HSV-1/2 ASR (Roche Diagnostics, Indianapolis)
Miller et al [38]	United States	Prospective	HSV-1, HSV-2	179	Genital herpes	IDbox HSV-1/2 assay (GenturaDx)	Molecular assays for detection of HSV	Culture; MultiCode HSV PCR
Parra-Sánchez et al [39]	Spain	Prospective	HSV-1, HSV-2	283	Various mucosal and cutaneous lesions, CSF, urinary	HSV OligoGen kit (Operon-Immuno&Molecular Diagnostics, Zaragoza, Spain)	Molecular assays for detection of HSV	Roche LightCycler HSVóQual Kit assay (Roche Diagnostics, Basel, Switzerland)
Patel et al [26]	South Africa and Zambia	Retrospective	HSV-2	3408	HSV-2/HIV-1 coinfected individuals and HIV-uninfected heterosexual partners	Kalon HerpeSimplex virus type 2 IgG ELISA (Kalon Biological Ltd, Surrey, United Kingdom)	Serology-based HSV detection assay	Washington HSV western blot
Van Der Pol et al [40]	United States	Prospective	HSV-1, HSV-2	508	Anogenital infections (anogenital)	BD ProbeTec HSV-Qx (HSVQx) system (BD Diagnostics, Sparks, MD)	Molecular assays for detection of HSV	Elvis culture system (San Diego); Laboratory-developed PCR assay
Shevlin and Morrow [41]	United States	Retrospective	HSV-2	100	Not specified	Uni-Gold HSV-2 rapid (Trinity Biotech, Ireland)	Serology-based HSV detection assay	Washington HSV western blot
Shoji et al [27]	Japan	Prospective	Viral subtype could not be ascertained	59 + 23 healthy volunteers	Herpes simplex keratitis (ocular)	HSV DNA (by PCR) and HSV specific sIgA antibody levels (ELIZA) in tears	Serology-based HSV detection assay and molecular assays for detection of HSV	—^h^ Control patients?
Tong et al [42]	United States	Prospective	HSV-1, HSV-2	176	Genital and oral lesions (oral and genital)	IsoGlow HSV typing assay (BioHelix Corp, Beverly, MA)	Molecular assays for detection of HSV	IsoAmp HSV (BioHelix Corp, Beverly, MA); ELVIS Shell Vial Assay (Diagnostic Hybrids, Athens, OH)

^a^HSV: herpes simplex virus.

^b^IgG: immunoglobulin G.

^c^ELISA: enzyme-linked immunosorbent assay.

^d^MAb-EIA: monoclonal antibody enzyme immunoassay.

^e^PCR: polymerase chain reaction.

^f^DFA: direct fluorescent antibody.

^g^LDT: laboratory developed test.

^h^Not applicable.

**Table 2:** Methodological characteristics of included studies, summarizing study aims, conclusions, and demographic data.

Study ID	Patient demographic data	Aims/rationale	Conclusions
Al‐Shobaili et al [24]	58% male; mean age 37 (SD 13.6) years	To evaluate a point-of-care test (HerpeSelect Express Rapid Test) for more rapid turnaround of results in a nonlaboratory setting.	The HerpeSelect Express Rapid Test has adequate sensitivity and specificity for confirming HSV^a^-2 infection in patients with genitourinary disease. The test is good at diagnosing high-risk individuals.
Barrado et al [28]	52.2% male; mean age 56.9 (SD 19.7) years	Assess if conjunctival swab samples were equivalent to corneal scrapings to diagnose herpetic keratitis.	Conjunctival swabs may serve as supplemental method for the diagnosis of typical HK^b^ despite limited sensitivity when the collection of corneal scrapings would not be feasible. PCR^c^ must be considered the gold standard for diagnosis of typical HK.
Burton et al [30]	92% male; 67% African American; 33% white; 97% heterosexual	To assess type-specific tests for HSV-2 in patients with chronic hepatitis C infection	In veterans with chronic hepatitis C infection, HerpeSelect 1 HSV-2 index values between 1.1 and 2.89 should be confirmed with an alternate test for HSV-2 infection. Only effective within certain range.
Gitman et al [31]	26.4% male; 148 adult; 23 pediatric	Compare the performance, time to result, and cost of the Simplexa HSV 1 & 2 Direct PCR with those of conventional	Simplexa HSV 1 & 2 Direct PCR is expensive but required the least training, had the lowest hands-on time and fastest assay time (75 min vs 3 hours by LDT^d^ HSV PCR), detected most positives, considered an internal control, and provided the HSV type.
Granato et al [32]	—^e^	To evaluate the performance of Amplivue HSV-1 + 2 assay compared with ELVIS HSV IDD3 in detecting HSV-1-2 from clinical specimens.	The results of this study show that the AmpliVue HSV-1/2 assay was more sensitive than ELVIS culture for detecting HSV-1 and HSV-2 in a wide variety of cutaneous and mucocutaneous specimens
Hobbs et al [33]	198 adult male and female; 18 samples used for Kalon assay; 178 samples used for the Focus assay	To evaluate and validate the use of Focus and Kalon ELISAs^f^ in detecting HSV-2 antibodies from dried blood sample as part of an HIV prevention trial.	The use of dried blood spots can reduce time and provide an effective way to test for HSV-2 in resource-limited settings. The study found that the use of dried blood spots with the Kalon assay did not perform well and that the use of dried blood spots with the Focus assay resulted in comparable sensitivity and specificity to the use of serum sample.
Lang et al [34]	—	Compare HSV-Q and real-time HSV PCR in terms of accuracy and cost. Device approved for anogenital infections; this study investigates its use on lesions from other anatomical locations.	The HSV-Qx assay was found to be highly sensitive and accurate. Gray zone may be required for specimens with values falling between 50 and 800 maximum relative fluorescence units. HSV-Qx has a lower cost per specimen ($22) compared with that of HSV-LC ($34). Samples lying near the positivity cut off should be retested.
Lee at al [35]	—	To evaluate the analytical and clinical performance of the Luminex ARIES assay in detecting HSV-1 and HSV-2 compared with an approved assay for the HSV testing for the diagnosis of meningitis/encephalitis.	The analytical performance results of the assay showed that the assay had lower sensitivity than the comparator. On the other hand, the clinical performance results of the assay showed that it had comparable performance to the routinely used assay. Compared with 2 other routinely used assays, the ARIES assay required the shortest amount of hands-on and assay time and was the least labor-intensive. The study concluded that Luminex ARIES assay can be used for successful detection of HSV-1 and HSV-2 for skin and genital infections, meningitis, and encephalitis.
Liermann et al [36]	Healthy children aged between 5 months and 3 years, healthy voluntary blood donors aged 18 to 65 years, or hospitalized patients aged between 14 and 70 years	Evaluate the HSV type-common and type-specific IgG^g^ and IgM^h^ enzyme-linked immunosorbent assays for the diagnosis of acute and latent HSV infections.	HSV type-common IgM ELISAs can be useful to confirm acute newly acquired HSV infections; the use of single-type IgM ELISAs on the basis of whole-virus antigen is dispensable.
Liu et al [37]	64% male; mean age 35.9 (SD 4.52) years for males; mean age 30.7 (SD 4.65) years for females	Need for a convenient, high-quality, rapid, and inexpensive domestic serodiagnostic kit that differentiates between HSV-1 and HSV-2.	The study indicates that gG_321–580H_ has a high diagnostic potential for HSV-2 virus serodiagnosis in humans.
Loughman et al [25]	30% male; median age of 40.5 (range: 15-85)	To validate the HSV-2 biochip assay as a point-of-care test to be able to use it in resource-limited settings.	Membrane-touch biochip requires some improvements such as expanded calibration before it can be used as a rapid diagnosis tool. The tool was validated and performs comparable to other standard diagnostic methods. The study concluded that the assay could work potentially as a rapid, point-of-care test for HSV-2.
Binnicker et al [29]	—	Investigates a more rapid, cheaper method of detecting HSV in CSF^i^ fluid that only uses a small amount of fluid. This makes it suitable for use in neonates.	The Simplexa HSV-1/2 assays demonstrated a combined sensitivity and specificity of 96.2% and 97.9%, respectively. Assay does not require nucleic acid extraction. Results are available in 60 min. The Simplexa assay requires only 50 mL of CSF.
Miller et al [38]	—	New system designed to maximize sensitivity. This study assesses whether this is achieved and is fully automated.	Assay shows acceptable clinical performance characteristics and demonstrates promise for further development of this fully automated platform for detection of pathogen nucleic acid in clinical laboratories
Parra-Sánchez et al [39]	42.2% male; median age 30.5 (Q1-Q3 24.0-39.0) years	Evaluate a new assay, the HSV OligoGen Kit for the detection of HSV in several types of clinical samples	Detection of HSV-1 and HSV-2 in clinical samples by HOK^j^ was not significantly different from detection by LCHK assay (*P* ≥.8, *t* test). Statistical data obtained in this study confirm the usefulness and reliable results of this new assay from a variety of specimens.
Patel et al [26]	51% male; median age (IQR) of 32.0 (27-39); HIV-positive: 50%	Evaluate an onsite HSV tool in a population of HIV infected and noninfected patients and to evaluate the precision of the Kalon HSV-2 IgG ELISA.	In populations with optimal diagnostic accuracy, Kalon is a reliable stand-alone method for on-site HSV-2 IgG antibody detection. Kalon can be utilized in resource-limited settings, enhancing the feasibility to monitor the epidemic and assess intervention efforts.
Van Der Pol et al [40]	34% male; median age: 25 years (range: 17-71)	Results of a multicenter study evaluating the performance of a recently FDA^k^-approved, commercially available, type-specific nucleic acid amplification test that allows type-specific HSV discrimination.	Assay performs as well as the other assays on a fully automated system that provides results within a few hours rather than many days. No differences in test performance based on gender, clinic type, location of the lesion, or type of lesion were observed.
Shevlin and Morrow [41]	35% male (estimate); Pediatric sera (age <18 years) were not included	Evaluation of a point-of-care and inexpensive device to detect anti-HSV anybody in serum or blood.	UHR is a reliable, low-cost alternative to other point-of-care HSV-2 diagnostic tests. Showed both sensitivity and specificity in a small group of adults.
Shoji et al [27]	51% male	Assesses whether the combined utilization of IgAantibody (HSV-sIgA) levels in tears and real-time PCR can improve diagnostic ability.	The combination of laboratory detection of HSV DNA by real-time PCR and of HSV-sIgA by ELISA using tear samples enables higher reliability in diagnosing the subgroups of HSK^l^.
Tong et al [42]	—	Substantial market need for a low-cost, point-of-care HSV typing assay. Such a device is assessed within this study.	Both formats of the IsoGlow HSV typing assay had sensitivities comparable to that of the FDA-cleared IsoAmp HSV test and specificity for the 2 types of HSV comparable with that of ELVIS HSV turnaround time of around 1 hour.

^a^HSV: herpes simplex virus.

^b^HK: Herpes Keratitis.

^c^PCR: polymerase chain reaction.

^d^LDT: laboratory developed test.

^e^Not applicable.

^f^ELISA: enzyme-linked immunosorbent assay.

^g^IgG: immunoglobulin G.

^h^IgM: immunoglobulin M.

^i^CSF: cerebrospinal fluid.

^j^HOK: HSV OligoGen kit.

^k^FDA: Food and Drug Administration.

^l^HSK: Herpes Simplex Keratitis.

**Table 3:** Molecular assays for the detection of herpes simplex virus, summarizing the different molecular assays studied within the 10 included studies. Information includes regulatory status, collection/storage/transport method, and performance.

Test	Study ID	Manufacturer	FDA^a^ status	EMA^b^ status	Sample type	Collection method
Real-time singleplex PCR^c^	Barrado et al [28]	University Hospital Madrid, Spain (academic)	No	No	Corneal scrapings and conjunctival swabs	Corneal scrapings by a platinum Kimura spatula using as viral transport medium UTM (Universal transport medium, Copan Diagnostic, Inc). Conjunctival swabs were obtained by polyester swabs.
Simplexa HSV 1 & 2 Direct	Gitman et al [31]	Focus Diagnostics, Cypress, CA	Yes	Unclear	Dermal, ocular, mouth	Swabs were collected from local clinics, doctors’ offices, and inpatient wards in 3 mL of M4 viral transport medium (Remel, Lenexa, KS)
Simplexa HSV 1 & 2 Direct	Binnicker et al [29]	Focus Diagnostics, Cypress, CA	Yes	Unclear	CSF^d^	Unknown how CSF collected.
AmpliVue HSV-1 & 2 assay	Granato et al [32]	Quidel, San Diego, CA	Yes	Unclear	Cutaneous and mucocutaneous swabs	All specimens were collected on swabs, transported to the laboratory in viral transport medium
Viper HSV-Qx assay	Lang et al [34]	BD Diagnostics, Sparks, MD	Yes (only for anogenital lesions)	Unclear	Anogenital and oral swabs	—^e^
Luminex ARIES HSV-1 & 2 assay	Lee et al [35]	Luminex Corp, Austen, TX	Yes (only for cutaneous or mucocutaneous lesion samples)	Unclear	Lesion swab samples	Lesion swab samples were collected in clinic and deidentified for the study
BD ProbeTec HSV-Qx (HSVQx) system	Van Der Pol et al [40]	BD Diagnostics, Sparks, MD	Yes (only for anogenital lesions)	—	Anogenital swabs	Sampled using a polyester swab, which was placed directly into universal viral transport medium (Becton, Dickinson [BD], Sparks, MD), and then using a second, foam-tipped swab, which was placed directly into the BDQx liquid wet-swab transport medium (Qx WS).
IDbox HSV-1/2 assay	Miller et al [38]	GenturaDx, Hayward, CA	No	No	Genital swabs	Swabs collected in 3 mL of universal transport medium, transported to labs, aliquoted samples stored in −70°C
HSV OligoGen kit	Parra-Sánchez et al [39]	Operon-Immuno&Molecular Diagnostics, Zaragoza, Spain	No	No	Various swabs; 110 ulcer specimens, 48 urine, 48 endocervical, 43 CSFs, 4 urethral, and 3 pharyngeal swabs	Cobas PCR media urine sample kit or cobas PCR media female swab sample kit (Roche Diagnostics GmbH, Mannheim, Germany); 110 ulcer specimens, 48 urine, 48 endocervical, 43 CSF, 4 urethral, and 3 pharyngeal swabs
HSV DNA (by PCR) and HSV specific sIgA antibody levels (ELIZA)	Shoji et al [27]	School of Medicine, Tokyo, Japan (academic)	No	No	Tears	Using Schirmer strips (Schirmer Tear Production Measuring Strips; Showa- Yakuhinkako, Tokyo, Japan)
IsoGlow HSV typing assay	Tong et al [42]	BioHelix Corp, Beverly, MA	No	Unclear	Swabs	Clinical swabs suspended in vial transport medium were collected from the Cleveland Clinic (Cleveland, OH). The samples were shipped on ice for overnight delivery and were aliquoted on receipt. Some were placed at −80°C for long-term storage, and some were placed at −20°C for short-term storage or near-term testing.

^a^FDA: Food and Drug Administration.

^b^EMA: European Medicines Agency.

^c^PCR: polymerase chain reaction.

^d^CSF: cerebrospinal fluid.

^e^Not applicable.

#### Serological Assays

**Figure 3:**
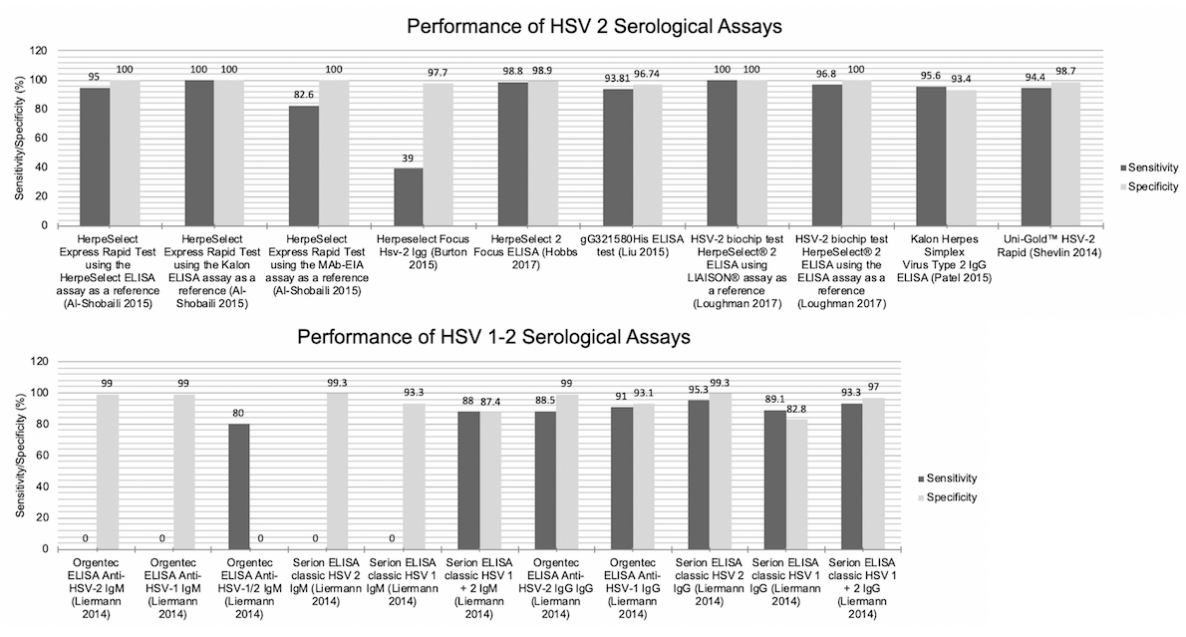
Sensitivity and specificity of molecular-based diagnostics. Because of the heterogeneous nature of studies included in this systematic review, it is not possible to draw strict conclusions with regard to performance metrics. This figure, therefore, aims solely to act as a means of visually displaying the sensitivity and specificity results of each study. HSV: herpes simplex viruses.

**Figure 4:**
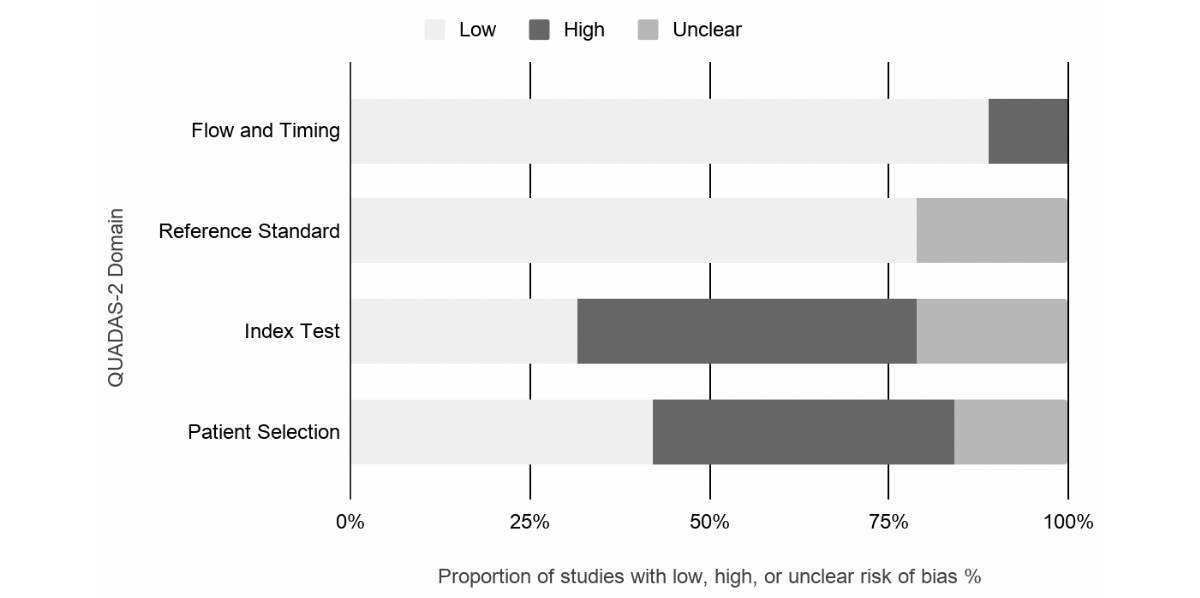
Graphical display for the Quality Assessment of Diagnostic Accuracy Studies-2 tool results. QUADAS: quality assessment of diagnostic accuracy studies.

Serological assays for the detection of HSV are summarized in Multimedia Appendix 3. A total of 19 serological diagnostic tools were investigated within 8 studies (plus 1 study [27] that used both molecular and serological methods in conjunction—detailed in Table 3); 4 of the serological diagnostic tools have received FDA approval (Multimedia Appendix 3) of which HerpeSelect 2 [33] was approved for use with serum only, and the test used by Loughman et al [25] only used reagents from the FDA-approved HerpeSelect 2 IgG kit. All of the studies in this category used serum samples, and only 1 study also tested the use of dried blood spots instead [33]. The performance metrics of the various tests are summarized for comparison to future technologies (Multimedia Appendix 3 and Figure 3). Sensitivity for serological tools ranged from 39% in hepatitis-coinfected individuals using the Herpeselect Hsv-2 IgG [30] tool to 100% (for a number of tools; Figure 4). All others were equal to or above 80% (Multimedia Appendix 3 and Figure 3). The study by Liermann et al [36] investigated the detection of primary infection and concluded that its HSV IgM serology tool should not be used to make decisions for antiviral treatment in HSV.

### Cost

Gitman et al [31] carried out the most comprehensive cost analysis, comparing the performance, time to result, and cost of the Simplexa HSV 1 & 2 Direct PCR with those of conventional cell culture, DFA, and a laboratory-developed real-time TaqMan PCR (LDT HSV PCR). The results of this are displayed in Table 4 (table was taken directly from the corresponding article [31]).

**Table 4:** Comparison of estimated costs per reportable result and expertise required. Table adapted from Gitman et al [31].

Test	Assay time	Frequency of testing	Cost ($)			Expertise
			Materials and reagents	Labor	Total	
Cytospin-DFA^a^	90 min	On demand	5.14	8.78	13.92	Mid-high
Conventional culture	1-7 days	Examined once a day	5.4	11.94	17.94	Mid-high
Simplexa Direct PCR	75 min	Once a day	39.54	2.69	42.23	Mid
LDT^b^ HSV^c^ PCR^d^ with extraction	3 h	Once a day	19.96	5.07	25.03	High

^a^DFA: direct fluorescent antibody.

^b^LDT: laboratory developed test.

^c^HSV: herpes simplex virus.

^d^PCR: polymerase chain reaction.

Gitman et al [31] concluded that the Simplexa HSV 1 & 2 Direct PCR was the most expensive but required the least training of the assays used, had the lowest hands-on time, and fastest assay time (75 min, vs 3 hours by LDT HSV PCR), and provided the HSV type. The Simplexa Direct PCR is an FDA-approved device (Table 3), and such could be used to benchmark developing technologies.

The cost-to-run per specimen was also compared between the Viper platform and real-time PRC in the study by Lang et al [34]. Compared with the HSV-LC system, the Viper instrument was found to be $12 cheaper to run per specimen ($22 vs $34). In addition, the platform is fully automated [34]. Although they did not carry out a formal cost analysis, the study by Binnicker et al [29] compared time with run for real-time PCR assay and the commercially available Simplexa HSV 1 & 2 Direct (Focus Diagnostics, Cypress, CA) using CSF samples. In addition, Lee et al [35] has reported that the automated ARIES assay takes only 2 hours/12 samples and has the fastest turnaround time compared with other in-house assays. In addition, to avoiding the need for nucleic acid extraction, the Simplexa devices run time was quoted as 60 min in comparison to 4 hours by conventional methods. A separate tool, the HSV-Qx assay, was found to produce results in a *few hours* compared with *many days* for conventional culture techniques [40]. Tong et al [42] also demonstrated a turnaround time of 1 hour using their portable device, although it was not fully automated (requires hands on time of 5 min).

### Sample Type and Patient Subpopulations

Binnicker et al [29] found that the Simplexa HSV-1/2 assays demonstrated a combined HSV-1 and HSV-2 sensitivity and specificity of 96.2% and 97.9%, respectively. Critically, the results are available in 60 min, and the test only requires 50 µL of CSF [29].

### Risk of Bias in Individual Studies

Most of the studies received high risk of bias in the patient selection and in the index test domains of the QUADAS tool (Table 5; Figure 4). High risk of bias in the patient selection domain was mostly because of the lack of randomization in recruiting patients and the use of case-control design in selecting participants and/or recruited patients with *confirmed diagnosis*. Only few studies included patients with suspected disease and no pretested samples [40,41] or recruited a random sample [26]. High risk of bias in the index test category was mostly because of the index test results being interpreted with the knowledge of the reference test results; most of the studies used the reference test first, and after knowing the results, performed the index test. Because all of the studies used the gold standard or an approved and validated reference tests, the reference standard mostly received low risk of bias (Figure 4). Studies that received high bias rating in the flow and timing domain used different reference standards for different patient groups and did not include all patients or samples in the analysis [27,36]. Studies that received low risk of bias rating in general recruited a random sample [26], blinded study conductors to the results of the reference and index tests [26,29,36], deidentified previously collected samples [41], or conducted reference and index tests simultaneously [34]. All of the studies received low risk of bias in the applicability concerns domains of the QUADAS tool because of the reviews’ inclusion criteria being inclusive of a wide range of patients, index tests, and references standards (Table 5).

**Table 5:** Tabular presentation for the Quality Assessment of Diagnostic Accuracy Studies tool results. Table format adapted from Whiting et al [22].

Study	Risk of bias				Applicability concerns		
	Patient selection	Index test	Reference standard	Flow and timing	Patient selection	Index test	Reference standard
Al-Shobaili et al [24]	Low risk	Unclear risk	Unclear risk	Low risk	Low risk	Low risk	Low risk
Barrado et al [28]	Low risk	High risk	Low risk	Low risk	Low risk	Low risk	Low risk
Burton et al [30]	High risk	Unclear risk	Unclear risk	Low risk	Low risk	Low risk	Low risk
Gitman et al [31]	Low risk	High risk	Low risk	Low risk	Low risk	Low risk	Low risk
Granato et al [32]	Low risk	Low risk	Low risk	Low risk	Low risk	Low risk	Low risk
Hobbs et al [33]	High risk	High risk	Low risk	Low risk	Low risk	Low risk	Low risk
Lang et al [34]	Unclear risk	Low risk	Low risk	Low risk	Low risk	Low risk	Low risk
Lee et al [35]	Low risk	High risk	Low risk	Low risk	Low risk	Low risk	Low risk
Liermann et al [36]	Unclear risk	Low risk	Low risk	High risk	Low risk	Low risk	Low risk
Liu et al [37]	High risk	Unclear risk	Low risk	Low risk	Low risk	Low risk	Low risk
Loughman et al [25]	High risk	High risk	Low risk	Low risk	Low risk	Low risk	Low risk
Binnicker et al [29]	High risk	Low risk	Low risk	Low risk	Low risk	Low risk	Low risk
Miller et al [38]	High risk	High risk	Low risk	Low risk	Low risk	Low risk	Low risk
Parra-Sánchez et al [39]	High risk	High risk	Low risk	Low risk	Low risk	Low risk	Low risk
Patel et al [26]	Low risk	Low risk	Low risk	Low risk	Low risk	Low risk	Low risk
Van Der Pol et al [40]	Low risk	Unclear risk	Unclear risk	Low risk	Low risk	Low risk	Low risk
Shevlin and Morrow [41]	Low risk	Low risk	Low risk	Low risk	Low risk	Low risk	Low risk
Shoji et al [27]	High risk	High risk	Unclear risk	High risk	Low risk	Low risk	Low risk
Tong et al [42]	Unclear risk	High risk	Low risk	Low risk	Low risk	Low risk	Low risk

## Discussion

### Overview

To select the most appropriate test for a patient, a thorough knowledge of the performance and limitations of available tests is critical to inform clinical decision making and the development of future technologies that may penetrate innovation gaps. This systematic review aimed to summarize recent study articles evaluating HSV-1 and HSV-2 diagnostic tools. We evaluated the study characteristics and the performance of the various tests included in the studies; here, we conclude by discussing the various strengths and limitations of various tools and how they may relate to the development of future diagnostics.

### Performance

It is evident that further work needs to be done with regard to tools that work better with different sample types. It also appears that tools developed in academic centers perform worse. This may be because they are at an earlier stage of the developmental pathways and thus, not nearing approval or commercialization. Overall, tests were more specific than sensitive, and it appears that serological tests perform worse.

Culture- and laboratory-based methods have become the gold standard choice for the diagnosis of HSV-1 and HSV-2 infections. However, translational effort is focused on developing alternate techniques based on serological and molecular assays as evidenced by the absence of novel culture-based methods in this review.

Specimen quality and adequate transport and handling to maintain infectivity are essential while using culture methods. Because of the enveloped nature of the HSV-1 and HSV-2 virus, specimens collected using a swab must be transferred to suitable viral transport media with the addition of antimicrobials. Using standard culture methods, results are usually available within 5 days; although in some cases, the result may take up to 2 weeks. Such issues are largely mitigated when using molecular methods. The use of an antigen detection system such as Herpchek (DuPont, Wilmington, DE) can decrease turnaround time [44]. Shell vial culture also has a decreased average turnaround time, with high sensitivity achieved within 24 hours [45]. In Tong et al [42], the IsoGlow HSV Typing assay (Nucleic acid amplification tests—based technique) was compared with the ELVIS Shell Vial Assay where it displayed similar performance, while having the advantages that it was more rapid, portable, and does not require laboratory equipment. Another limitation of traditional culture methods is that viral subtyping requires the additional step of using HSV-1 and HSV-2 monoclonal antibodies [46,47]. Similarly Lee et al [35], found that the ARIES assay required the least amount of time when compared with 2 other routinely used assays and the least technical skills and knowledge [35]. The enzyme-linked virus-inducible system (ELVIS; Diagnostic Hybrids, Inc, USA) enables faster turnaround than standard culture or shell vial methods, with similar performance characteristics [45]. High sensitivities using culture-based methods are hindered by highly variable specimen quality, dependency on stage of the lesion, as well as primary versus recurrent infection [47].

### Turnaround Time and Cost

In HSV infections, especially when it results in CNS-related infections, rapid turnaround is essential for treatment [48,49]. Rapid turnaround is important for providing the treatment to patient as early as possible. In the case of HSV, which has no cure, antivirals are the only treatment available to manage the symptoms of the disease [50]. Antivirals are recommended to be administered within the first 48 to 72 hours of the appearance of symptoms for best results [51], which is a very short time interval that requires a rapid turnaround time in case the provider has to wait for the diagnostic test results before prescribing the medication. Even more important is the throughput time (ie, how many tests can be run using the system within certain amount of time) of a diagnostic test. In most cases, a confirmed diagnostic is necessary to provide antiviral treatment for HSV since the prolonged use of antivirals can lead to antiviral resistance [50]. According to Lee et al [35], the ARIES assay’s throughput time was reported as 12 samples in 2 hours. This throughput time is rapid compared with 3 to 7 days of processing time needed for viral culture [52].

Automation increases turnaround time, improve reproducibility of the test, and reduce the chance of human error [53]. Although not quantified, capital would be saved because of the automated nature and more rapid turnaround of such devices. Automated diagnostic tests may only require minimal technical knowledge reducing the likelihood or *economically wasteful misclassification* because of limited knowledge of the lab technician [53]. Rapid turnaround time is important for most infectious or viral diseases. For example, for respiratory viruses, diagnostic test should be provided at the point-of-care (possible with results ready in less than 1.6 hours) to ensure the best outcomes for the patient [54].

### Sample Type and Patient Subpopulations

The HIV status of an individual does not appear to affect the performance of HSV ELISAs [55]. This is an important point because of the high rate of coinfection and increased risk of transmission and acquisition of HIV by HSV-positive individuals [56,57]. Among other common sexually transmitted diseases, only coinfection with *Neisseria gonorrhoeae* has been shown to significantly reduce HSV immunoassay’s performance [57]. However, the specificity of immunoassays can differ based on population demographics based on the findings of a meta-analysis conducted on studies from Africa [13].

While HSV can rarely be cultured from the CSF, molecular assays can yield results in a matter of hours, which can be important when dealing with possible HSV encephalitis or meningitis. Currently, the only device approved for use for HSV virus detection in CSF is the Simplexa HSV 1 & 2 Direct (Focus Diagnostics, Cypress, CA) (Table 4). The latter is important for the use of the test in the neonatal population. Neonates immune system has a limited ability in fighting off infections, hence resulting in mortality rates exceeding 80% from HSV infection [58]. A PCR-based HSV detection system from CSF is critical for this patient population. Given this information, it is concerning that greatest incidence of HSV infections occurs in women of reproductive age increasing the risk of maternal transmission of the virus to the fetus or neonate [59,60]. This is a major public health concern in both developed and developing countries: a 3-year study in Canada found a neonatal HSV incidence of 5.9 per 100,000 live births and a case fatality rate of 15.5% [61]. Prevention is challenging given the asymptomatic nature of the disease in its latent phase [61], and current guidelines recommend delivery by cesarean section. This is because 85% of vertical transmissions occur during the vaginal delivery where the fetus comes into contact with infected genital secretions [61]. A 33-year study conducted in the New York, USA, concluded that deaths from HSV have increased over the time period (incidence 0.82 deaths per 100,000 live births), despite reductons in deaths from HIV and syphilis [62]. They stated this is due to an increasing number of pregnant women having no immunity to HSV type 1 putting them at increased risk of contracting the disease during pregnancy [62]. Interestingly, neonatal HSV-1 infection in developing countries is rare with the vast majority of infections being caused by HSV-2, which carries a worse prognosis. In addition, acute infection is associated with a higher transmission rate in part because of the lack of maternal to fetal antibody transfer in the third trimester [60]. A test that can accurately diagnose acute (not just latent infection) is urgently required, as current methods may not detect early infection. The study by Liermann et al [36] was the only study to investigate detection of primary infection, and concluded that its HSV IgM serology tool should not be used to make decisions for antiviral treatment in HSV [36]. Thus, no diagnostic tools included in this systematic review have addressed this unmet medical need.

The use of serologic tests to diagnose suspected genital lesions are inappropriate because positive results may be due to chronic infection, whereas negative results may overlook recent infection.

### Risk of Bias of the Included Studies

A common source of bias in diagnostic tool studies occurs when studies recruit patients based on their disease status. This often leads to overestimation of the results and may exaggerate diagnostic accuracy [22]. All of the studies included in this review used sample populations that had a higher than average prevalence of HSV infection. As a result, these sensitivities may be overestimated.

### Limitations

The studies included within this systematic review employed heterogeneous methods while also using different patient populations. For this reason, a statistical comparison of results was not conducted, and instead, the results displayed in a qualitative manner to benchmark future technology. Although a PRISMA-compliant search was searching the PubMed database, it is possible that some studies may have been inadvertently missed.

### Conclusions

The landscape of diagnostic tools for HSV-1 and HSV-2 infections is rapidly moving away from laboratory-based and culture methods that have long been considered the gold standard technique. A majority of tools study cutaneous and mucosal HSV infections (n=13); 2 tests focused on ocular disease, whereas a single one on CNS manifestations. No diagnostic tools included in our systematic review are currently suitable for use as prenatal tools, however. The incidence of acute infections is rising, and because these infections present the greatest risk to unborn fetuses, further work needs to be done to develop diagnostic tools to detect primary infection in expectant mothers to prevent vertical transfer. This will assist in lowering the rate of neonatal herpes, which can be a life-threatening condition. We believe this can only be achieved through prenatal screening for primary infection and subsequent medical intervention.

## Appendix

Multimedia Appendix 1 Search terms.

Multimedia Appendix 2 Preferred Reporting Items for Systematic Reviews and Meta-Analyses 2009 flow diagram.

Multimedia Appendix 3 Serological assays for detection of herpes simplex virus and summary of the different serological assays studied within the 6 included studies.

## Abbreviations

CNS: central nervous system
CSF: cerebrospinal fluid
DFA: direct fluorescent antibody
ELISA: enzyme-linked immunosorbent assay
FDA: Food and Drug Administration
HSV: herpes simplex viruses
PICO: Population, Intervention, Comparator, Outcome
PRISMA: Preferred Reporting Items for Systematic Reviews and Meta-Analyses
QUADAS: quality assessment of diagnostic accuracy studies
