# Supplementary material for: Tools for the Diagnosis of Herpes Simplex Virus 1/2: Systematic Review of Studies Published Between 2012 and 2018
Source: JMIR Public Health Surveill. 2019 May 23;5(2):e14216. doi: 10.2196/14216 (PMC6552407; doi:10.2196/14216)
Supplement: Multimedia Appendix 1 [file publichealth_v5i2e14216_app1.docx]

**Multimedia Appendix 1**

**Search Terms**

| **PubMed** |
| --- |
| (((((( "Simplexvirus/genetics"[Mesh] OR "Simplexvirus/isolation and purification"[Mesh] )) OR ( "Herpes Simplex/diagnosis"[Mesh] OR "Herpes Simplex/virology"[Mesh] )) OR ( "Herpesvirus 2, Human/genetics"[Mesh] OR "Herpesvirus 2, Human/isolation and purification"[Mesh] )) OR ( "Herpesvirus 1, Human/genetics"[Mesh] OR "Herpesvirus 1, Human/isolation and purification"[Mesh] )) OR ( "Herpes Genitalis/diagnosis"[Mesh] OR "Herpes Genitalis/virology"[Mesh] )) AND "Sensitivity and Specificity"[Mesh] |
| (("Polymerase Chain Reaction"[Mesh]) OR "Multiplex Polymerase Chain Reaction/methods"[Mesh]) OR "Real-Time Polymerase Chain Reaction/methods"[Mesh]) OR "Fluorescent Antibody Technique/methods"[Mesh]) OR "Nucleic Acid Amplification Techniques/methods"[Mesh]) OR "Blotting, Western/methods"[Mesh]) OR "Enzyme-Linked Immunosorbent Assay"[Mesh]) AND ("Herpes Simplex/diagnosis"[Mesh] OR "Herpes Simplex/virology"[Mesh] ) |
