# Supplementary material for: Tools for the Diagnosis of Herpes Simplex Virus 1/2: Systematic Review of Studies Published Between 2012 and 2018
Source: JMIR Public Health Surveill. 2019 May 23;5(2):e14216. doi: 10.2196/14216 (PMC6552407; doi:10.2196/14216)
Supplement: Multimedia Appendix 2 [file publichealth_v5i2e14216_app2.doc]

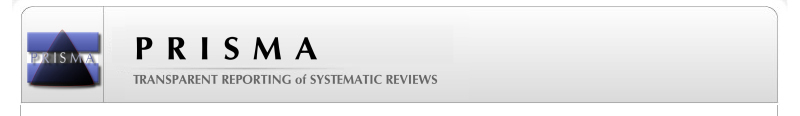
**PRISMA 2009 Flow Diagram**

**Screening**

**Included**

**Eligibility**

**Identification**

Records identified through database searching
(n=264)

Additional records identified through other sources
(n=30)

Records after duplicates removed
(n=264)

Records screened
(n=264)

Records excluded
(n=209)

Full-text articles assessed for eligibility
(n=59)

Full-text articles excluded, with reasons
(n=40)

- Does not state sensitivity/or specificity of test (n=8)
- No performance metric stated (n=8)
- Study concerns viruses in addition to HSV1 and/or HSV2 (n=11)
- Article is a review (n=4)
- Research article does not concern a diagnostic tool (n=9)

Studies included in qualitative synthesis
(n=19)
