# Supplementary material for: Tools for the Diagnosis of Herpes Simplex Virus 1/2: Systematic Review of Studies Published Between 2012 and 2018
Source: JMIR Public Health Surveill. 2019 May 23;5(2):e14216. doi: 10.2196/14216 (PMC6552407; doi:10.2196/14216)
Supplement: Multimedia Appendix 3 [file publichealth_v5i2e14216_app3.docx]

Multimedia Appendix 3. Serological assays for detection of herpes simplex virus and summary of the different serological assays studied within the 6 included studies. Information includes regulatory status, collection/storage/transport method, and performance.

| Test TTest | Study ID | Manufacturer | FDA^a^ status | EMA^b^ Status | Sample type | Collection Method | Transportation/Storage Method | Performance % (95% CI) | | | | | | |
| --- | --- | --- | --- | --- | --- | --- | --- | --- | --- | --- | --- | --- | --- | --- |
|  |  |  |  |  |  |  |  | Sensitivity | Specificity | PPV^c^ | NPV^d^ | Agreement | Disconconrdant results | Other metrics |
| HerpeSelect Express Rapid Test | Al-Shobaili et al [24] | Focus Technologies Inc., Cypress, CA | Yes | Unclear | Serum | Blood samples collected in pyrogen-free tubes under aseptic conditions. Allowed to be clotted and centrifuged. | Frozen in aliquots at –20^o^C until use. | 95 when compared with HerpeSelect ELISA^e^; 100 when compared with Kalon ELISA; 82.6 when compared with mAb-EIA^f^ | 100 when compared with HerpeSelect ELISA; 100 when compared with Kalon ELISA; 100 when compared with mAb-EIA | 100 when compared with HerpeSelect ELISA; 100 when compared with MAb-EIA | 97.56 when compared with HerpeSelect ELISA; 100 when compared with Kalon ELISA; 90.24 when compared with mAb-EIA | 98.3 when compared with HerpeSelect ELISA; 100 when compared with Kalon ELISA; 93.3 when compared with mAb-EIA | Potentially 5 | Able to deal with 5 equivocal results |
| HerpeSelect Focus Hsv^g^-2 IgG^h^ | Burton et al [30] | Focus Technologies Inc., Cypress, CA | Yes | Unclear | Serum | Archived samples | N/A^i^ | 39.0 (24.2–55.5) | 97.7 (87.7–99.6) | 94.1 (71.2–99.0) | 62.7 (50.0–74.2) | N/A | N/A | N/A |
| HerpeSelect 2 Focus ELISA | Hobbs et al [33] | Focus Diagnostics, Cypress, CA | Yes (for use with serum only) | Unclear | Serum (dried blood spots) | Blood samples obtained through venipuncture and stored in EDTA^j^ containing tubes. Dried blood spot samples were prepared by spotting EDTA blood onto S&S 903 filter paper (Schleicher & Schuell, Dassel, Germany), dried in a biosafety cabinet for 18-24 hours, and stored at 4^o^C until use | Dried blood spots eluted with phosphate buffered saline at 4°C for 18-24 hours | 98.8 (92.7-99.9) | 98.9 (93.4-99.9) | N/A | N/A | Unweighted Kappa: 0.96 (0.913-0.999) | N/A | N/A |
| Kelon ELISA | Hobbs et al [33] | Kalon Biological, Guildford, United Kingdom | No | Unclear | Serum (dried blood spots) | Blood samples obtained through venipuncture and stored in EDTA containing tubes. Dried blood spot samples were prepared by spotting EDTA blood onto S&S 903 filter paper (Schleicher & Schuell, Dassel, Germany), dried in a biosafety cabinet for 18-24 hours, and stored at 4^o^C until use | Dried blood spots diluted with a kit diluent | <9.1 (0-25.9) | 100 (64.6-100) | N/A | N/A | Unweighted Kappa < 0.068 (0- 0.369) | N/A | N/A |
| Orgentec ELISA Anti-HSV-2 IgM | Liermann et al [36] | Orgente, Mainz, Germany | No | No | Serum | N/A | Stored in aliquots at −20°C | Not calculated | 99.0 (93.8-99.9) | N/A | N/A | N/A | Cannot quantify discrepant results | N/A |
| Orgentec ELISA Anti-HSV-1 IgM | Liermann et al [36] | Orgente, Mainz, Germany | No | No | Serum | N/A | N/A Stored in aliquots at −20°C | Not calculated | 99.0 (93.8-99.9) | N/A | N/A | N/A | Cannot quantify discrepant results | N/A |
| Orgentec ELISA Anti-HSV-1/2 IgM | Liermann et al [36] | Orgente, Mainz, Germany | No | No | Serum | N/A | N/A Stored in aliquots at −20°C | 80.0 (58.7-92.4) (cut-off 12.5 U/mL) | Not tested | N/A | N/A | N/A | Cannot quantify discrepant results | N/A |
| Serion ELISA classic HSV-2 IgM | Liermann et al [36] | Serion, Würzburg, Germany | No | No | Serum | N/A | N/A Stored in aliquots at −20°C | Not calculated | 99.3 (95.8-100.0) to 100.0 (96.9-100.0) | N/A | N/A | N/A | Cannot quantify discrepant results | N/A |
| Serion ELISA classic HSV-1 IgM | Liermann et al [36] | Serion, Würzburg, Germany | No | No | Serum | N/A | N/A Stored in aliquots at −20°C | Not calculated | 93.3 (87.8-96.6) to 96.0 (91.2-98.4) | N/A | N/A | N/A | Cannot quantify discrepant results | N/A |
| Serion ELISA classic HSV-1 + 2 IgM | Liermann et al [36] | Serion, Würzburg, Germany | No | No | Serum | N/A | N/A Stored in aliquots at −20°C | 88.0 (67.7-96.8) to 96.0 (77.7-100.0) | 87.4 (80.8-92.1) to 94.7 (89.5-97.5) | N/A | N/A | Kappa: 0.96 (0.88-1.00) | Cannot quantify discrepant results | N/A |
| Orgentec ELISA Anti-HSV-2 IgG IgG | Liermann et al [36] | Orgente, Mainz, Germany | No | No | Serum | N/A | N/A Stored in aliquots at −20°C | 88.5 (79.4-94.1) to 95.4 (88.0-98.5) | 99.0 (93.8-99.9) | N/A | N/A | Kappa: 0.94 (0.90-0.99) | Cannot quantify discrepant results | N/A |
| Orgentec ELISA Anti-HSV-1 IgG | Liermann et al [36] | Orgente, Mainz, Germany | No | No | Serum | N/A | N/A, stored in aliquots at −20°C | 91.0 (83.2-95.5) to 96.0 (89.5-98.7) | 93.1 (85.0-97.2) to 100.0 (94.7-100.0) | N/A | N/A | Kappa: 0.95 (0.91-1.00) | Cannot quantify discrepant results | N/A |
| Serion ELISA classic HSV-2 IgG | Liermann et al [36] | Serion, Würzburg, Germany | No | No | Serum | N/A | N/A, stored in aliquots at −20°C | 95.3 (87.7-98.5) to 97.6 (91.0-99.6) | 99.3 (95.8-100) to 100.0 (96.9-100.0) | N/A | N/A | Kappa: 0.98 (0.96-1.00) | Cannot quantify discrepant results | N/A |
| Serion ELISA classic HSV-1 IgG | Liermann et al [36] | Serion, Würzburg, Germany | No | No | Serum | N/A | N/A, stored in aliquots at −20°C | 89.1 (81.0-94.2) to 98.0 (92.3-99.7) | 82.8 (75.1-88.6) to 97.0 (92.0-99.0) | N/A | N/A | Kappa: 0.93 (0.88-0.98) | Cannot quantify discrepant results | N/A |
| Serion ELISA classic HSV-1 + 2 IgG | Liermann et al [36] | Serion, Würzburg, Germany | No | No | Serum | N/A | N/A, stored in aliquots at −20°C | 93.3 (87.4-96.7) to 95.6 (90.2-98.2) | 97.0 (90.8-99.2) to 99.0 (93.8-99.9) | N/A | N/A | Kappa: 0.94 (0.89-0.98) | Cannot quantify discrepant results | N/A |
| gG_321580His_ ELISA test | Liu et al [37] | Hangzhou Center for Disease Control and Prevention, Hangzhou, China (academic) | No | No | Serum | N/A | Sera were frozen and stored at –70°C | 93.81 | 96.74 | N/A | N/A | N/A | N/A | Overall accuracy - 94.65%, No false identification of HSV-1. |
| HSV-2 biochip test HerpeSelect 2 ELISA | Loughman et al [25] | Focus Diagnostics, CA | Only some components of the test | Unclear | Serum | N/A | —^k^ | Using LIAISON assay as reference: 100 using ELISA assay as reference: 96.8 | Using LIAISON assay as reference: 100 using ELISA assay as reference: 100 | N/A | N/A | N/A | — | — |
| Kalon Herpes Simplex Virus Type 2 IgG ELISA | Patel et al [26] | Kalon Biological Ltd, Surrey, United Kingdom | No | No | Serum | N/A | Serum samples were consented for long term storage in the US and were stored at −80°C | 95.6 (93.4-97.3) (cut-off=1.1) | 93.4 (86.9-97.3) (cut-off=1.1) | N/A | N/A | N/A | Of the 16 indeterminate samples by UW-WB^l^, 10 (62.5%) were positive by Kalon. No indeterminate samples by UW-WB or Kalon had symptoms of GUD^m^ in their medical history (past 3 months) or had physical presentation of GUD. | AUC: 0.95 (0.92-0.97) (cut-off=1.1) |
| Uni-Gold™ HSV-2 Rapid | Shevlin and Morrow [41] | Trinity Biotech, Ireland | No | No | Serum | N/A | Frozen at −20°C | 94.4 | 98.7 | 94.4 | 98.7 | 98 | Uni-Gold HSV-2 Rapid gave a negative but valid test result for 3 sera that had nonspecific ban ding that could not be identified definitively as being related to HSV by UWWB | One false seronegative and one false seropositive result occurred. |
| HSV-sIgA by enzyme-linked immunosorbent assay (ELISA) | Shoji et al [27] | Division of Ophthalmology, Department of Visual Sciences, Nihon University School of Medicine | Unclear | — | Serum (tears) | Serum samples collected using the Schirmer I method from the lower fornix | HSV antigens added to wells with tear samples and incubated at room temperature for 1 h | 49.2 | 82.6 | — | — | — | — | — |

^a^DFA: direct fluorescent antibody.

^b^LDT: laboratory developed test.

^c^HSV: herpes simplex virus.

^d^PCR: polymerase chain reaction.
